# Comparative Evaluation of Bone Anatomy for Ramal Implant Placement and Proximity to the Inferior Alveolar Canal in Different Facial Divergence to Assess the Ideal Site of Placement: A Cone-Beam Computed Tomography Study

**DOI:** 10.7759/cureus.78290

**Published:** 2025-01-31

**Authors:** Aarohi Singh Rathor, Alap Shah, Bharvi Jani, Jhalak Vekariya, Manushi V Shah

**Affiliations:** 1 Orthodontics and Dentofacial Orthopaedics, Karnavati School of Dentistry, Gandhinagar, IND

**Keywords:** bone screws, disimpaction second molar, ramal implants, temporary anchorage devices (tads), uprighting second molar

## Abstract

Objective

The objective of this study is to determine the optimal location for placing the Ramal implant and assess the maximum transverse width of the ramal bone and the proximity of the implant to the inferior alveolar canal (IAC) through cone-beam computed tomography (CBCT) scans.

Materials and methods

The CBCT scans of 30 patients were utilized in this study and its proximity to the IAC at different vertical heights (3, 5, and 7 mm) and four angles of insertion (0°, 10°, 15°, and 20°). The maximum transverse width of the ramus and the proximity to the IAC from the site of insertion were measured at three different vertical levels.

Another important factor that was evaluated is the proximity of the Ramal implant to the IAC. After measuring the Ramal width, a central point was identified on it. From this point, a perpendicular line was drawn, which was parallel to the occlusal plane. Considering this line as a reference, angulations of 0°, 10°, 15°, and 20° were measured.

Results

The maximum transverse ramal width was seen in the hypodivergent growth pattern, i.e., 15.69 ± 0.952 mm at 3 mm. The highest clearance from the IAC was seen at a 20° angle in the hypodivergent growth pattern, which was 6.46 ± 2.76 mm at 3 mm.

Conclusion

The ramus of the mandible can be a predictable site for implant placement provided the variations in the anatomical structures have been carefully analyzed. It can be concluded that the Ramal implants can be safely placed at a level 3-7 mm above the permanent mandibular first molar above the occlusal plane and at 10°-15° in the case of hypo-divergent and normodivergent growth patterns at 20° or more in the case of a hyper-divergent growth pattern.

## Introduction

The core of orthodontics is attaining optimal anchorage without the unwanted movement of a reciprocal unit. Skeletal anchorage helps mitigate the undesirable consequences of orthodontic tooth movements by allowing the skeletal framework to absorb reactive forces.

Mini implants serve as invaluable sources of absolute anchorage. The envelope of discrepancy refers to the limitations of tooth movement within the dental arches without causing harm to the surrounding structures. Dr. Park recognized that by strategically placing temporary anchorage devices (TADs) for anchorage, orthodontists could effectively extend the envelope of discrepancy, enabling them to address more complex cases and achieve optimal treatment outcomes previously considered difficult to manage without surgery [1]. The optimal locations for mini-implant placement are thick and dense cortical bone regions [2]. Regions of the D1-D3 bone are adequate for TAD placement having enough compact bone during early stages, and the bone density appears to be a key determinant for stationary anchorage. Facial type is a determinant factor for comprehensive orthodontic treatment planning as the density and thickness of cortical bone are also affected by growth patterns [3]. Studies show that the buccal and lingual cortical plate thickness was greater in patients having hypo-divergent growth patterns than in normodivergent and hyper-divergent individuals [4,5].

Mini-implants have been effectively employed in achieving optimal dental movements within traditional treatment plans, such as molar protraction [6], retracting canines [7], correcting the dental midline [8], closing space [9], retracting maxillary incisors [10], distalizing maxillary molars [11], and uprighting second molars.

Mini-implants are a valuable tool for orthodontists, but managing impacted teeth presents significant challenges. Determining whether to upright or extract an impacted mandibular molar can pose a challenging decision during treatment planning. Reserving impacted teeth is preferred because natural dentition's durability and functional advantages, particularly its proprioception, cannot be replaced.

Impaction is most commonly observed in maxillary and mandibular third molars, with maxillary canines and mandibular second molars following closely in terms of relative incidence. The occurrence rate of impacted second molars is less than or equal to 2.3% [4,12].

Several methods involving mini-implants made of titanium placed in the middle of the roots of mandibular teeth to upright impacted molars have been proposed by Giancotti et al. [13] and others [5,14-23]. While these implants were effective in maintaining anchorage for space closure and opening, they had the disadvantage of high failure rates [24]. Furthermore, they were not specifically devised to tackle issues such as horizontally impacted mandibular molars. Tooth movement was hindered by these implants, the likelihood of mobility within the bone was increased, and there was a heightened potential for damage to tooth roots.

An extra-alveolar bone screw made up of stainless steel or titanium alloy having a diameter of 2 mm and a length of 14 mm was introduced for insertion into areas of dense cortical bone, like the anterior border of the ramus. In clinical settings, larger and longer mini-implants offer the benefit of enhanced primary stability and the capability to distribute applied force across larger bone areas, resulting in reduced bone stress [4,25,26].

Although Ramal implants have shown success in various clinical scenarios, concerns regarding their safety continue to be persistent. These issues stem from the variations in locations of neurovascular structures, the complex anatomy of the mandibular ramus, the thickness of soft tissue at the insertion place, difficulties in determining the precise insertion direction, and identifying the most favorable height above the occlusal plane for implant placement [4,26-30]. The main concern is the proximity to the inferior alveolar canal. The distance of the inferior alveolar canal to the anterior border of the ramus of the mandible varies among individuals and can also depend on the location along the ramus. Generally, the canal is located deep within the mandible, closer to its medial surface [31]. The muscular structure in the retromolar region consists of the traversing fibers of the medial pterygoid muscle and the anterior fibers of the temporalis muscle, both of which are attached to the surface of the ramus [4,32].

On average, the distance from the anterior border of the ramus to the inferior alveolar canal is approximately 10-15 mm [4]. However, this distance may vary based on factors such as age, gender, and individual anatomical variations such as facial divergence. The inferior alveolar nerve's position varies with age, gender, and anatomy. It is closer to the alveolar crest in children and edentulous elderly, with gender and anatomical variations affecting its location and surgical risks.

Therefore, the aim and objective of this research endeavor were to thoroughly investigate and identify the precise and optimal location for the insertion of Ramal implants by assessing the influence of facial divergence on the mandibular anatomy and evaluating the proximity of the implants to critical neurovascular structures, such as the inferior alveolar nerve and the associated vascular networks.

## Materials and methods

Study design and sample size

The study adopted a retrospective cross-sectional design, employing purposive sampling to select participants. The research investigated the cone-beam computed tomography (CBCT) pretreatment scans of 30 patients undergoing orthodontic treatment.

The power of the study was calculated to determine the sample size for the study. The sample size was determined using the following formula:

n = (Z^2 × σ^2) / E^2,

where n = sample size, Z = Z-value (e.g., 1.96 for 95% confidence level), 𝜎 = standard deviation of the population = 3, E = margin of error (desired precision) = 1.1. Based on this equation, the sample size comes out to be 29; therefore, we approximated it to be 30 samples.

The sample was stratified into three equal groups based on varying degrees of facial divergence on pretreatment lateral cephalogram for comparative analysis (N = 30, n = 10). The facial divergence patterns were categorized as hypodivergent (n = 10), normodivergent (n = 10), and hyperdivergent (n = 10) based on cephalometric measurements using three parameters, i.e., mandibular plane angle [33], Jarabak ratio [34], and Vert index [35]. These measurements were done on pre-orthodontic records, i.e., lateral cephalogram of all the patients.

The mandibular plane angle is the angle between the SN plane and mandibular plane (Go-Gn); normodivergent: 32°±5, hypodivergent: <27°, and hyperdivergent: >37°. Jarabak ratio is the posterior facial height (S-Go)/anterior facial height (N-Me) × 100; normodivergent: 62-65%, hypodivergent: >65%, hyperdivergent: <62%. Vert index is derived from Ricketts' five factors: severe hyperdivergent: ≤-2, light hyperdivergent: -1.9 to -0.5, normodivergent: -0.4 to +0.4, hypodivergent: +0.5 to +0.9, and severe hypodivergent: ≥+1.

With the approval of the ethical committee (KSDEC/22-23/Apr/017) at the Department of Orthodontics and Dentofacial Orthopaedics, Karnavati School of Dentistry in Gandhinagar, India, the CBCT scans were collected from May 2023 for three months, and the study was completed by April 2024.

The inclusion and exclusion criteria are as follows: Full-mouth scans were conducted on adult patients aged 18-35 years, regardless of gender, with a complete set of permanent teeth, a consistent mandibular occlusal plane, and no prior orthodontic treatment or surgical history. Patients were excluded if they had periapical pathologies, periodontal disease, bone loss, metabolic bone disorders, missing/extracted/supernumerary/mesioversion/impacted teeth, prostheses, craniofacial disorders, and facial asymmetries. Ethnicity was not taken into consideration.

CBCT Examinations

The CBCT data were obtained using a three-dimensional volume scanner (Genoray Papaya 3D+, South Korea). The scan data was stored in DICOM format. These files were then used to create 3D images, and measurements were recorded using CS 3D Imaging software (version 3.10, Carestream Health Inc., NY, USA).

The CBCT specifications are 120 kV, 5 mA exposure, 24s scan time, 300 µm voxel size, and 300 µm focal spot.

Skeletal Reference Point

**Figure 1:**
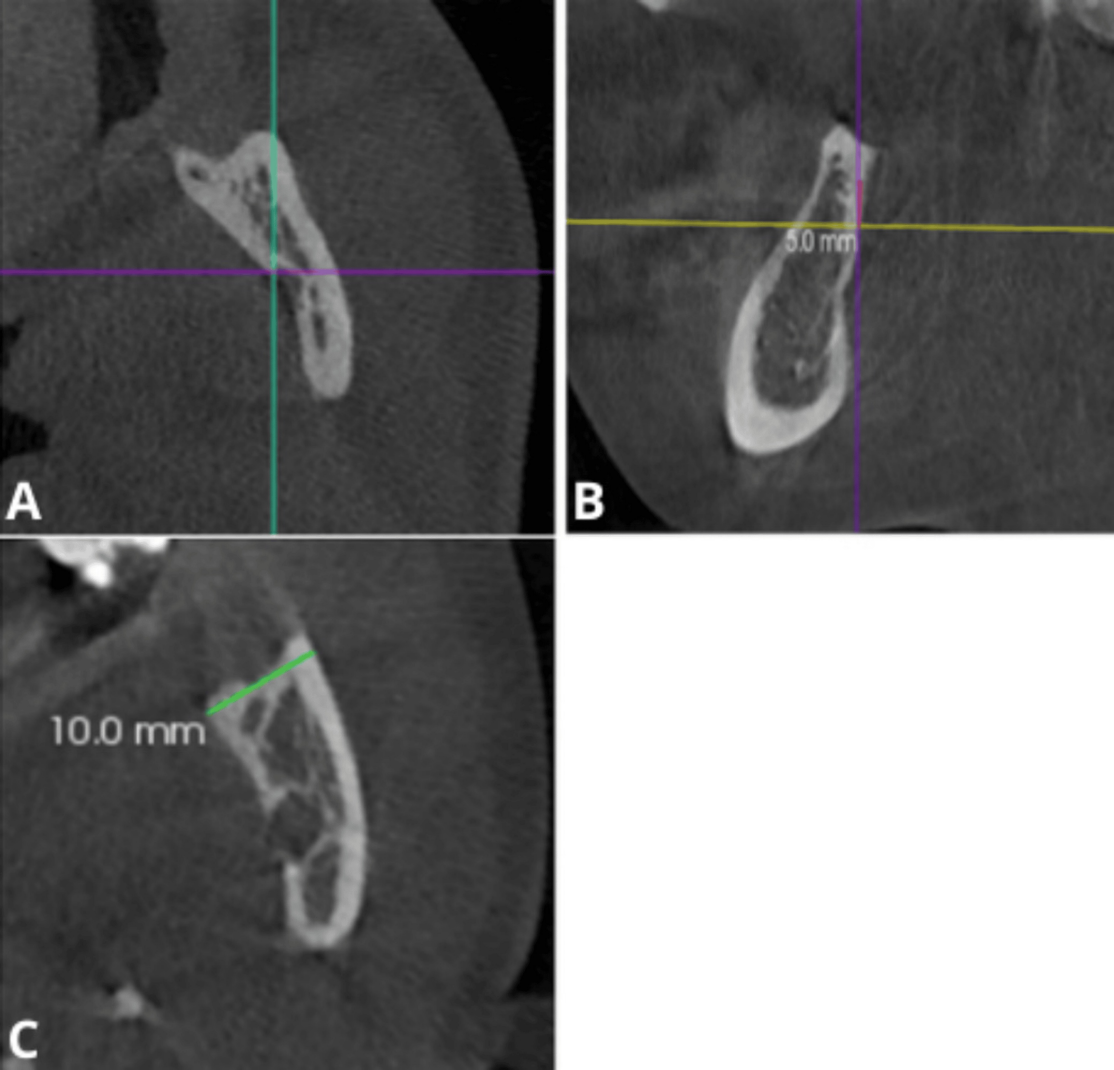
On the axial view of the cone-beam computed tomography (CBCT), the most prominent point of the lingula, considered the skeletal reference point, is identified. This point is located at the intersection of the green and purple lines (A). On the sagittal plane, a measurement of 5 mm is marked above the identified skeletal reference point to establish a new reference level (B). The axial slider is then adjusted to this level, and the maximum transverse width of the ramus is measured on the axial plane (C).

The lingula, a stable anatomical landmark, was used as a skeletal reference point to eliminate disparities caused by malocclusions affecting the occlusal plane. The axial plane was aligned to identify the most prominent point of the lingula (Figure 1).

Establishing the Reference Planes

**Figure 2:**
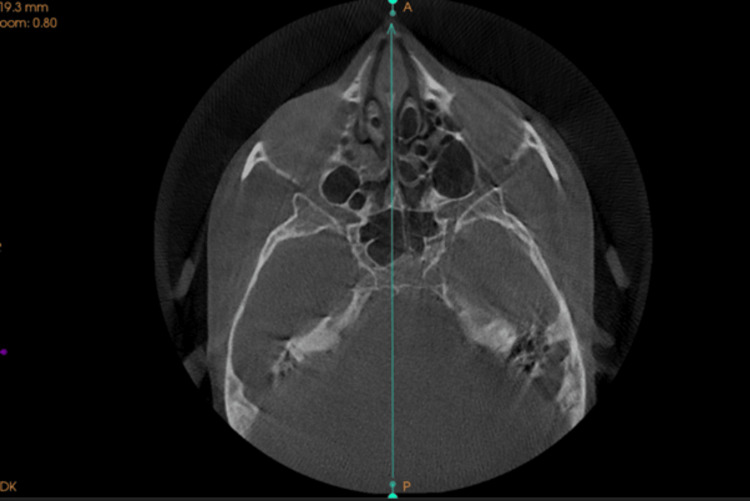
Mid-sagittal plane passing from cribriform plate, anterior nasal spine and opisthion.

To achieve consistent alignment of 3D images, the mandibular occlusal plane was designated as the horizontal reference plane. This plane was defined by connecting the tips of the mesiobuccal cusps of the first molars and the tip of the right central incisor in the mandibular arch. In addition, the midsagittal plane was established using anatomical landmarks such as the crista galli, anterior nasal spine (ANS), and opisthion (Figure 2).

Selection of Dental Reference Points for Ramal Bone Assessment

**Figure 3:**
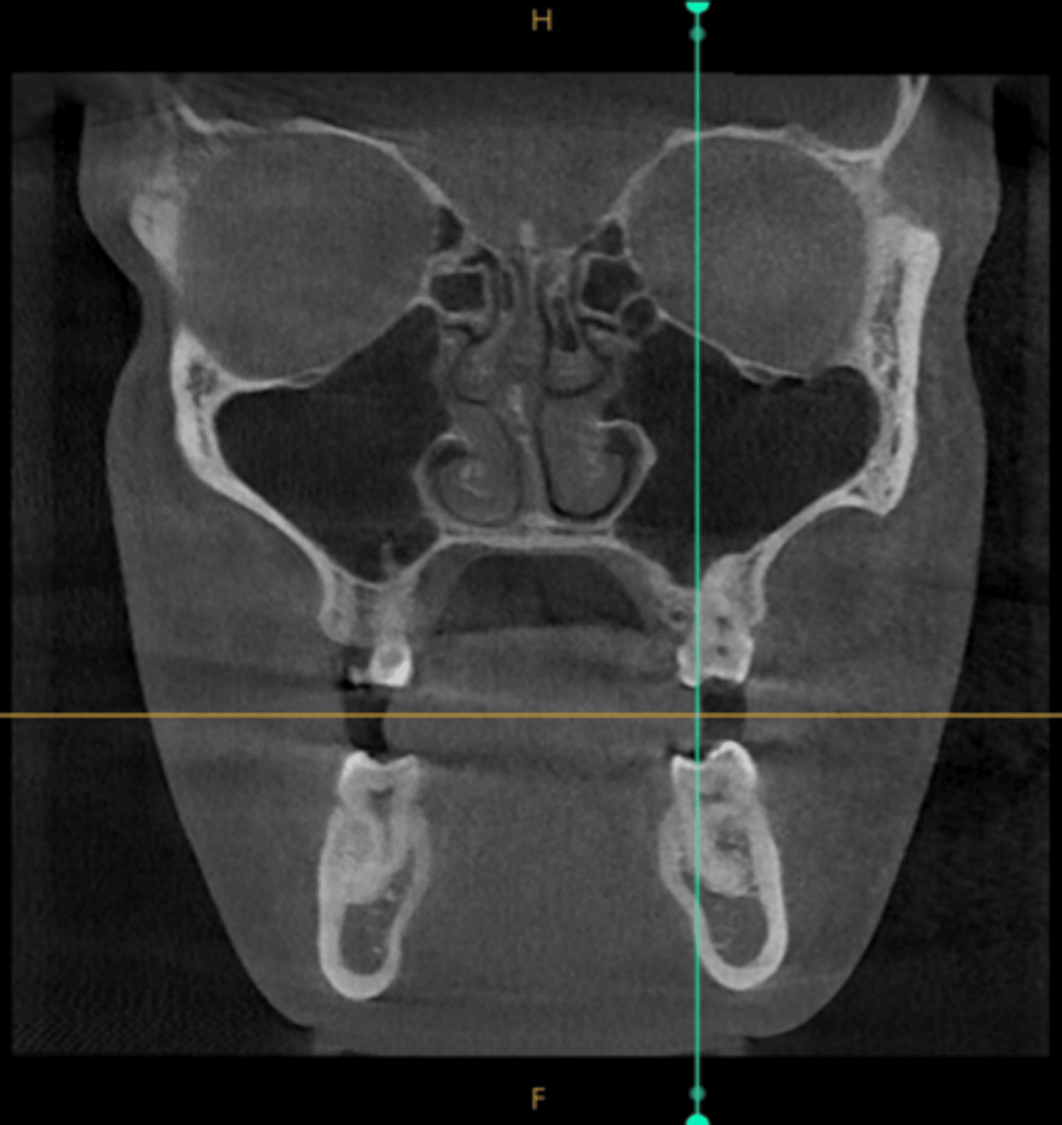
In the coronal view, the green line slider is aligned with the central groove of the mandibular first molar, serving as the dental reference point.

In this study, the central groove of the mandibular first molar was selected as the dental reference point to measure the maximum transverse width of the Ramal bone. The location of the mandibular first molar was established using the sagittal plane slider on the CBCT scan. The axial plane slider was then adjusted to align the axial plane with the level of the mandibular occlusal plane. The coronal plane was positioned over the central groove of the mandibular molar (Figure 3).

**Figure 4:**
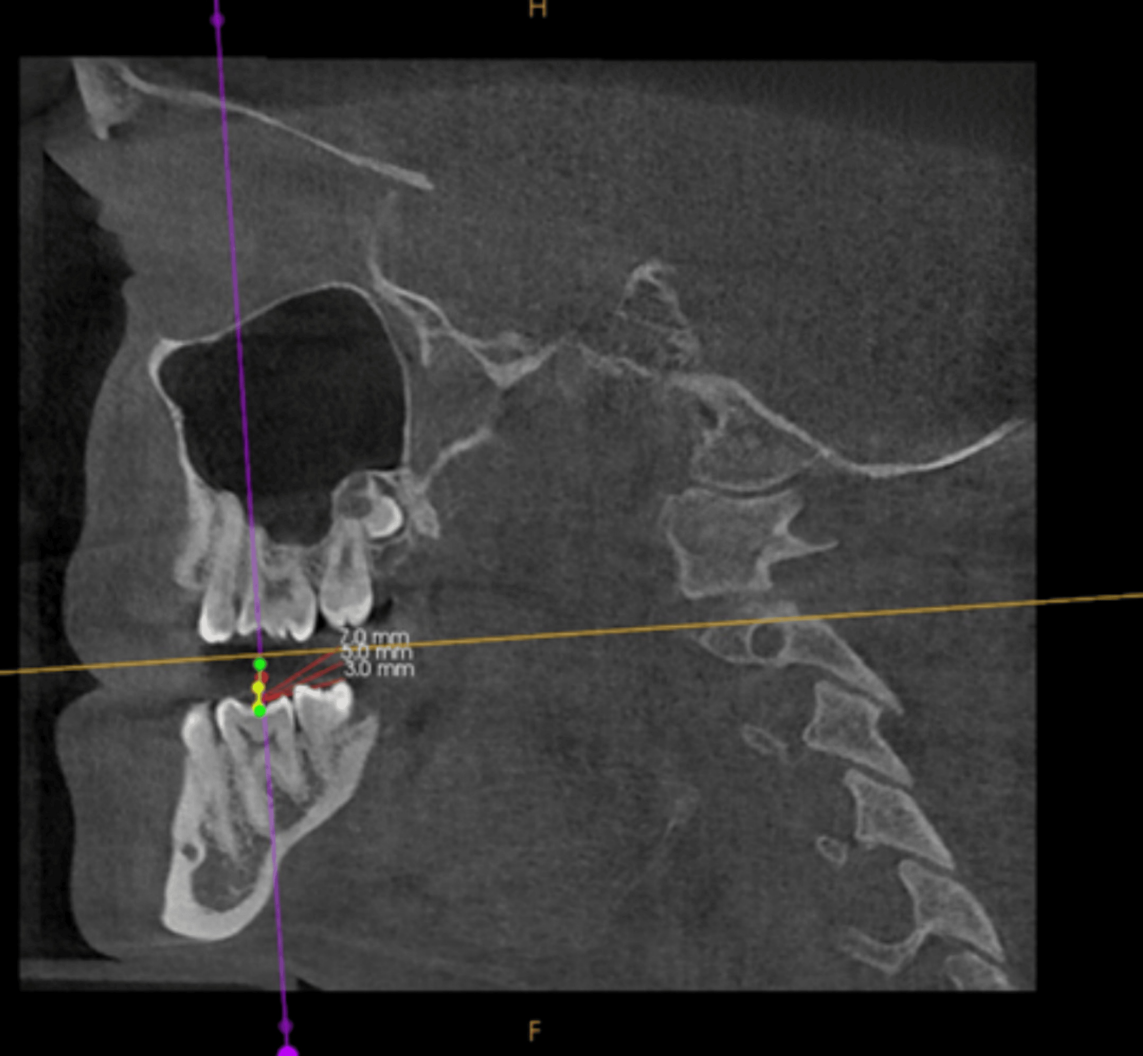
In the sagittal view, measurements of 3, 5, and 7 mm are taken perpendicular to the occlusal plane from the dental reference point.

The maximum transverse width of the Ramal bone was evaluated at three distinct vertical levels, i.e., 3, 5, and 7 mm, superior to the dental reference point within the axial plane. These levels were selected based on the recommendation that Ramal implants be placed at a level of 5 to 8 mm above the occlusal level (Figure 4). These measurements were performed only on the right side, due to bilateral symmetry.

Proximity of Ramal Implant to the Inferior Alveolar Canal (IAC)

After measuring the width of the Ramal bone, a central point was identified. From this point, a perpendicular line parallel to the occlusal plane was drawn. Using this line as a reference, angulations of 0°, 10°, 15°, and 20° were measured.

**Figure 5:**
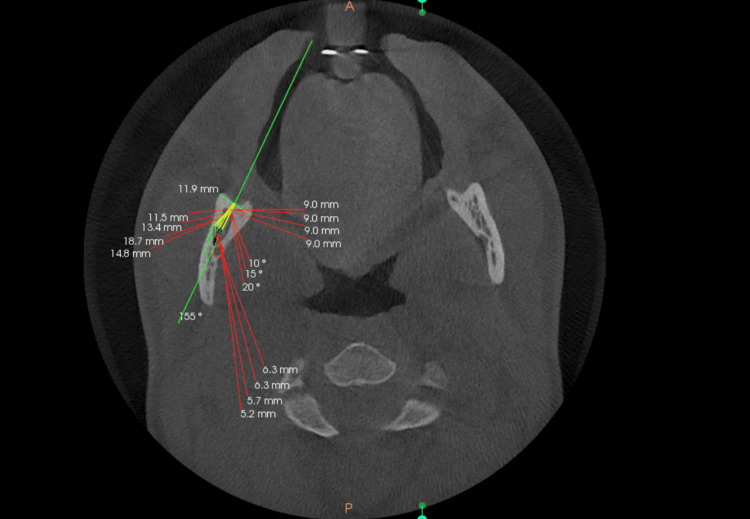
The axial plane is then adjusted to the specified level (yellow line), where the maximum transverse width of the ramus is measured. On the same axial plane, the midpoint of the measured maximum transverse ramal width is identified and selected as the insertion point for the ramal implant. A 5 mm implant length is used, and the distance to the inferior alveolar canal (IAC) is measured at four different insertion angles along this length.

A ramus implant requires traversing thicker soft tissue before reaching the dense cortical bone of the mandible. To ensure a 5 mm clearance for hygiene purposes, approximately 5 mm of bone engagement is possible. Considering the typical dimensions of a ramal implant (2 mm x 14 mm) and the soft tissue thickness (3-4 mm) surrounding the retromolar region, a 5 mm engagement of the ramal bone is facilitated. Therefore, markings were made at 5 mm from various angulations (0°, 10°, 15°, and 20°) at each level (3, 5, and 7 mm). Proximity to the inferior alveolar canal was assessed from these marked points (Figure 5).

Statistical analyses

Descriptive statistics and one-way analysis of variance (ANOVA) were done by a single operator to assess the variation in ramus width and its proximity to the IAC across the different levels. The significant level for the statistical analyses was set at p<0.05. All statistical analyses and graphical representations were performed using Stata 17 software (Stata 17, StataCorp LLC, College Station, Texas, USA).

## Results

Intragroup (ramus width at different vertical levels between the same growth patterns) and intergroup (ramus width at the same level between different growth patterns) comparisons were carried out.

Ramus width

Intragroup Comparisons

**Figure 6:**
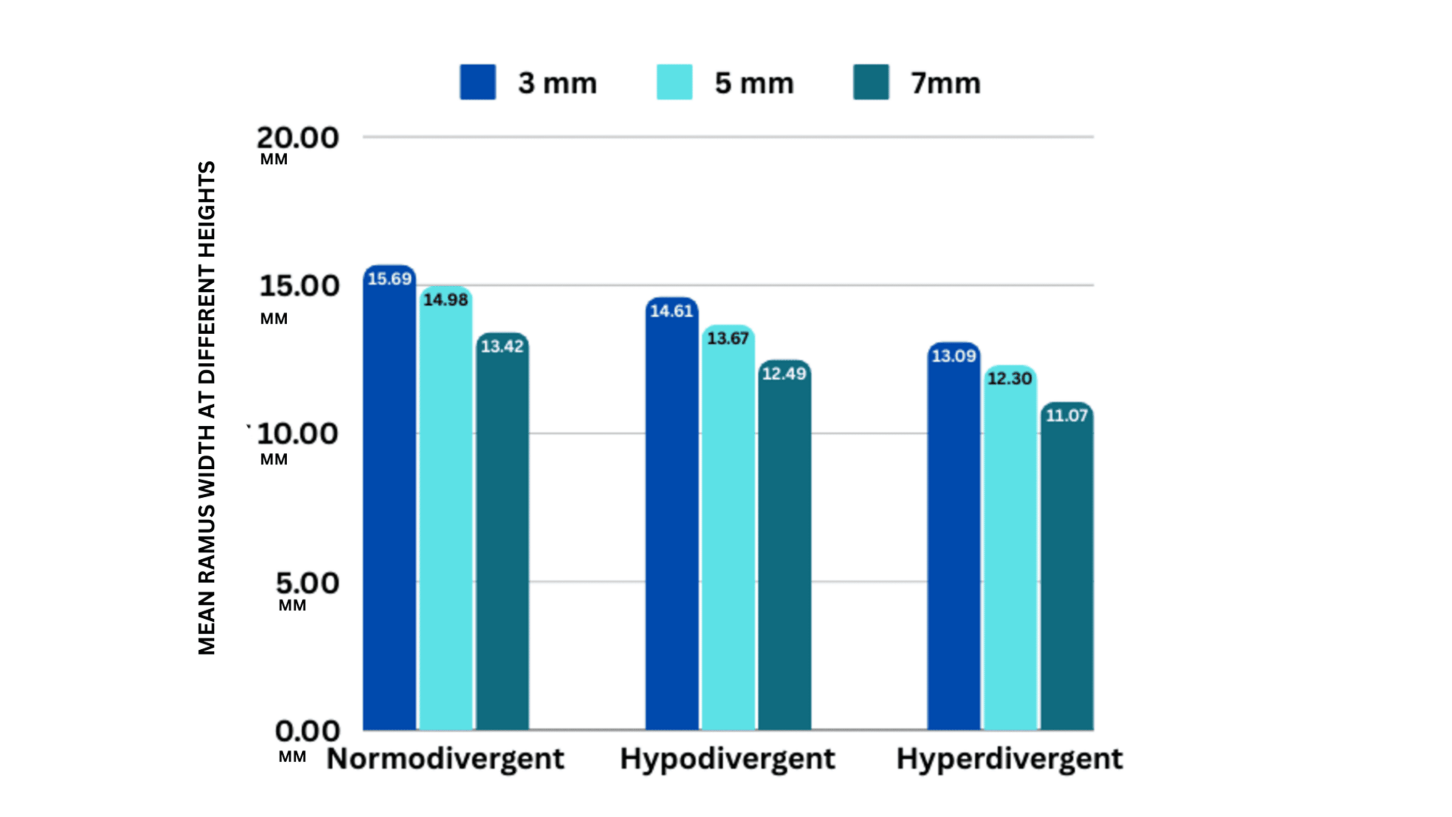
Intragroup comparison showing ramus width at different vertical levels between the same growth patterns

The maximum ramus width was observed at 3 mm for all growth patterns. For the normodivergent growth pattern, the ramus width is 14.61 ± 1.024 mm at 3 mm, 13.67 ± 0.742 mm at 5 mm, and 12.49 ± 1.008 mm at 7 mm (Figure 6, Table 1). In the hypodivergent growth pattern, the values are 15.69 ± 0.952 mm at 3 mm, 14.98 ± 0.623 mm at 5 mm, and 13.42 ± 1.441 mm at 7 mm. The widths for the hyperdivergent growth pattern are 13.09 ± 0.901 mm at 3 mm, 12.3 ± 0.039 mm at 5 mm, and 11.07 ± 1.096 mm at 7 mm. The one-way ANOVA shows significant differences across the growth patterns (p < 0.001).

**Table 1:** Intragroup comparison of ramus width at different levels between the same growth patterns

Growth pattern		n	Mean	Standard deviation	F	p-value
Normodivergent	3 mm	10	14.61	1.024	12.01	0.001*
	5 mm	10	13.67	0.742		
	7 mm	10	12.49	1.008		
Hypodivergent	3 mm	10	15.69	0.952	12.94	0.001*
	5 mm	10	14.98	0.623		
	7 mm	10	13.42	1.441		
Hyperdivergent	3 mm	10	13.09	0.901	17.06	0.001*
	5 mm	10	12.3	0.039		
	7 mm	10	11.07	1.096		

Intergroup Comparisons

The transverse ramus width is greatest in the hypodivergent growth pattern, followed by the normodivergent, and smallest in the hyperdivergent growth pattern at all levels (Table 2). The one-way ANOVA indicates significant differences across these growth patterns (p < 0.001).

**Table 2:** Tukey’s post-hoc analysis showing the pairwise comparison

Growth pattern	Comparing column I v/s column J		Mean difference (I -J)	p value
	(I)	(J)		
Hypodivergent	3 mm	5 mm	0.71	0.308
Hypodivergent	3 mm	7 mm	2.27	<0.001
Hypodivergent	5 mm	7 mm	1.56	0.008
Normodivergent	3 mm	5 mm	0.94	0.081
Normodivergent	3 mm	7 mm	2.12	<0.001
Normodivergent	5 mm	7 mm	1.18	0.023
Hyper divergent	3 mm	5 mm	0.81	0.076
Hyper divergent	3 mm	7 mm	2.14	<0.001
Hyper divergent	5 mm	7 mm	1.32	0.002

Tukey’s post-hoc analysis shows the pairwise comparison of the ramus width with the other. The groups are placed in two columns group (I) and group (J). The mean difference is seen in column (I-J). A negative value of mean difference indicates that the value in group (I) is less than that seen in group (J) and a positive value in the mean difference column suggests that the value in group (I) is more than that seen in group (J). 

In hypodivergent, the mean difference is highest between 3 mm and 7mm. There is a statistically significant difference between 3 mm and 7 mm and 5 mm and 7 mm. In normodivergent, the mean difference is the highest between 3 mm and 7 mm. There is a statistically significant difference between 3 mm and 7 mm and 5 mm and 7 mm. In hyperdivergent, the mean difference is the highest between 3 mm and 7 mm. There is a statistically significant difference between 3 mm and 7 mm and 5 mm and 7 mm.

Accurate angle of placement

3 mm

**Figure 7:**
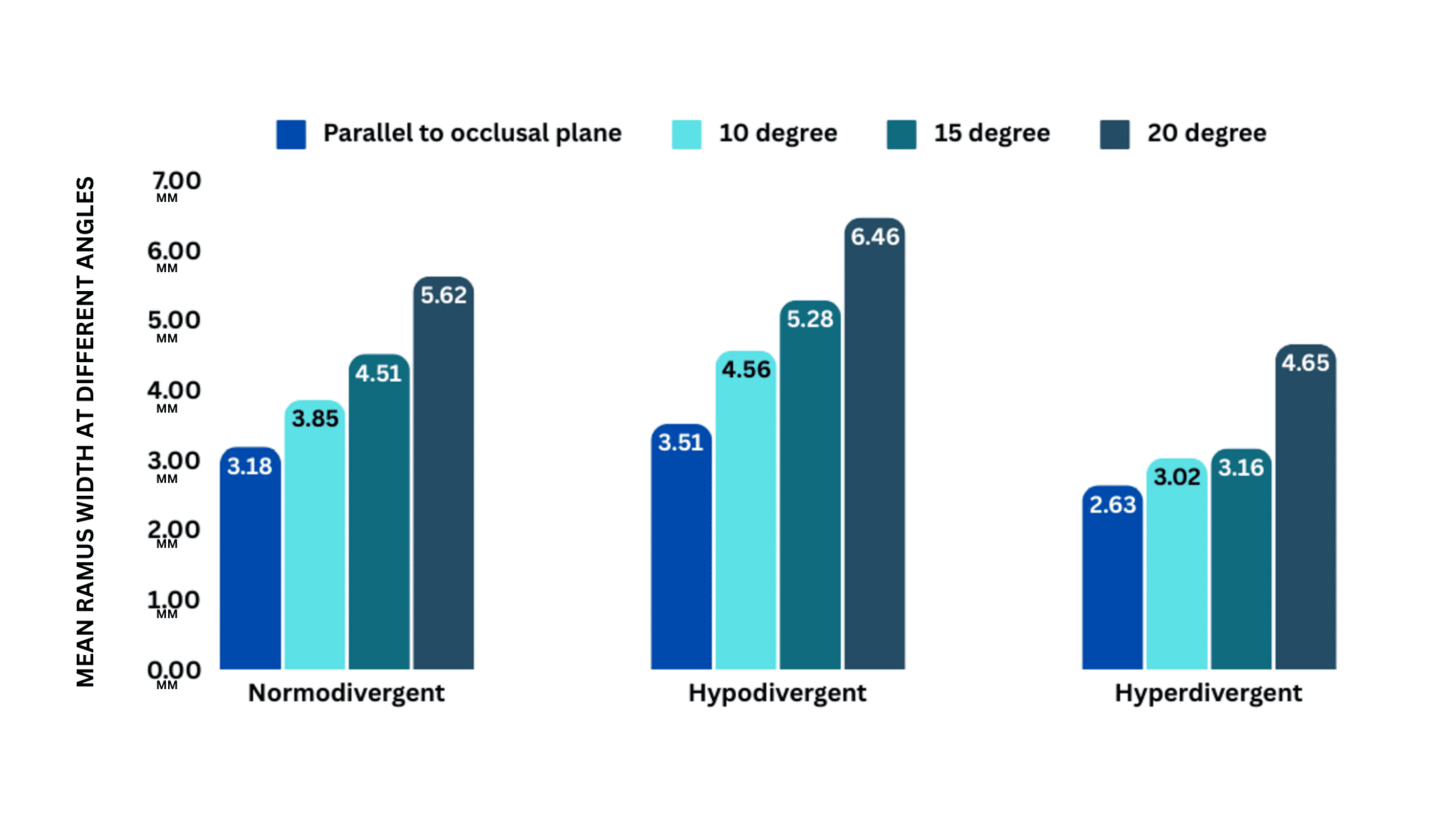
Intergroup comparison showing four angles of insertion in different growth patterns at 3 mm

No significant difference (p > 0.05) exists in angles across growth patterns. The highest clearance is observed at a 20° angle for all patterns: normodivergent (5.62 ± 1.85), hypodivergent (6.46 ± 2.76), and hyperdivergent (4.65 ± 2.31) (Figure 7, Tables 3-4).

**Table 3:** Intergroup comparison of angle of insertion with different growth patterns for 3 mm.

Growth pattern		Angle of insertion	N	Mean	Standard deviation	One-way ANOVA	
						F	p-value
Normodivergent	3 mm	Parallel to the occlusal plane	10	3.18	2.062	2.69	0.061
	3 mm	10 degree	10	3.85	2.07		
	3 mm	15 degree	10	4.51	2.029		
	3 mm	20 degree	10	5.62	1.849		
Hypodivergent	3 mm	Parallel to the occlusal plane	10	3.51	2.485	2.2	0.105
	3 mm	10 degree	10	4.56	2.564		
	3 mm	15 degree	10	5.28	2.757		
	3 mm	20 degree	10	6.46	2.767		
Hyperdivergent	3 mm	Parallel to the occlusal plane	10	2.63	2.123	1.5	0.23
	3 mm	10 degree	10	3.02	2.274		
	3 mm	15 degree	10	3.18	2.426		
	3 mm	20 degree	10	4.65	2.315		

**Table 4:** Tukey’s post-hoc test for the intergroup comparison for 3 mm

	Growth pattern (I)	Growth pattern (J)	Mean difference (I-J)	p-value
Hyperdivergent	10 degree	15 degree	-0.16	0.999
	10 degree	20 degree	-1.63	0.395
	10 degree	Parallel to the occlusal plane	0.4	0.979
	15 degree	20 degree	-1.47	0.485
	15 degree	Parallel to the occlusal plane	0.56	0.947
	20 degree	Parallel to the occlusal plane	2.03	0.213
Hypodivergent	10 degree	15 degree	-0.72	0.929
	10 degree	20 degree	-1.9	0.388
	10 degree	Parallel to the occlusal plane	1.05	0.811
	15 degree	20 degree	-1.18	0.752
	15 degree	Parallel to the occlusal plane	1.77	0.45
	20 degree	Parallel to the occlusal plane	2.95	0.078
Normodivergent	10 degree	15 degree	-0.66	0.882
	10 degree	20 degree	-1.77	0.216
	10 degree	Parallel to the occlusal plane	0.67	0.877
	15 degree	20 degree	-1.11	0.607
	15 degree	Parallel to the occlusal plane	1.33	0.458
	20 degree	Parallel to the occlusal plane	2.44	0.047

Tukey’s post-hoc analysis shows the pairwise comparison of the angle of insertion with the other for 3 mm in different growth patterns. The groups are placed in two columns group (I) and group (J). The mean difference is seen in column (I-J). A negative value of mean difference indicates that the value in group (I) is less than that seen in group (J), and a positive value in the mean difference column indicates that the value in group (I) is more than that seen in group (J). 

In hypodivergent, the mean difference is the highest between 20° and parallel to the occlusal plane. There is no statistically significant difference between difference in angles of insertion. In normodivergent, the mean difference is the highest between 20° and parallel to the occlusal plane. There is a statistically significant difference only between 20° and parallel to the occlusal plane. In hyperdivergent, the mean difference is the highest between 20° and parallel to the occlusal plane. There is no statistically significant difference between difference in angles of insertion.

5 mm

**Figure 8:**
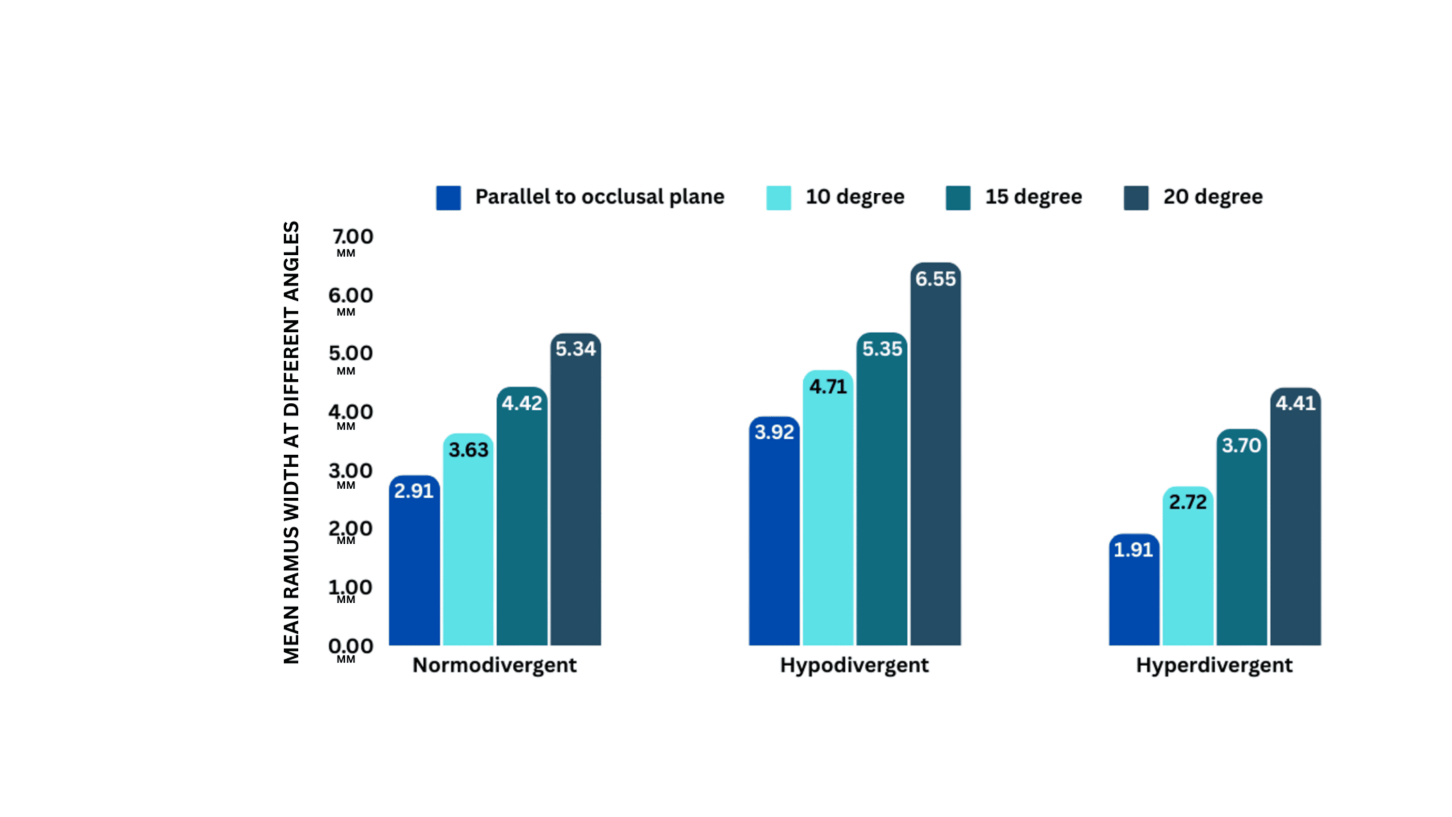
Intergroup comparison showing four angles of insertion in different growth patterns at 5 mm

There is a significant difference (p < 0.05) in angles across growth patterns. The highest clearance is at a 20° angle: normodivergent (5.34 ± 1.46), hypodivergent (6.55 ± 2.22), and hyperdivergent (4.41 ± 2.41) (Figure 8, Tables 5-6).

**Table 5:** Intergroup comparison of angle of insertion with different growth patterns for 5 mm.

Growth pattern		Angle of insertion	N	Mean	Standard deviation	One-way ANOVA	
						F	p-value
Normodivergent	5 mm	Parallel to the occlusal plane	10	2.91	1.791	4.53	0.008
		10 degree	10	3.63	1.397		
		15 degree	10	4.42	1.500		
		20 degree	10	5.34	1.468		
	Hypodivergent	5 mm	Parallel to the occlusal plane	10	3.92	2.382	2.57	0.069
		10 degree	10	4.71	2.259			
		15 degree	10	5.35	1.878			
		20 degree	10	6.55	2.221			
Hyperdivergent	5 mm	Parallel to the occlusal plane	10	1.91	1.919	2.53	0.073
		10 degree	10	2.72	2.166		
		15 degree	10	3.7	2.194		
		20 degree	10	4.41	2.412		

**Table 6:** Tukey’s post-hoc test for the intergroup comparison

	Growth pattern (I)	Growth pattern (J)	Mean difference (I-J)	p-value
Hyperdivergent	10 degree	15 degree	-0.98	0.747
	10 degree	20 degree	-1.69	0.322
	10 degree	Parallel to the occlusal plane	0.81	0.839
	15 degree	20 degree	-0.71	0.885
	15 degree	Parallel to the occlusal plane	1.79	0.274
	20 degree	Parallel to the occlusal plane	2.5	0.067
Hypodivergent	10 degree	15 degree	-0.64	0.914
	10 degree	20 degree	-1.84	0.256
	10 degree	Parallel to the occlusal plane	0.79	0.851
	15 degree	20 degree	-1.2	0.616
	15 degree	Parallel to the occlusal plane	1.43	0.473
	20 degree	Parallel to the occlusal plane	2.63	0.051
Normodivergent	10 degree	15 degree	-0.79	0.666
	10 degree	20 degree	-1.71	0.082
	10 degree	Parallel to the occlusal plane	0.72	0.727
	15 degree	20 degree	-0.92	0.55
	15 degree	Parallel to the occlusal plane	1.51	0.147
	20 degree	Parallel to the occlusal plane	2.43	0.006

Tukey’s post-hoc analysis shows the pairwise comparison of the angle of insertion with the other for 5 mm in different growth patterns. The groups are placed in two columns group (I) and group (J). The mean difference is seen in column (I-J). A negative value of mean difference indicates that the value in group (I) is less than that seen in group (J), and a positive value in the mean difference column suggests that the value in group (I) is more than that seen in group (J). In hypodivergent, the mean difference is highest between 20° and parallel to the occlusal plane. There is no statistically significant difference between difference in angles of insertion. In normodivergent, the mean difference is the highest between 20° and parallel to the occlusal plane. There is a statistically significant difference only between 20° and parallel to the occlusal plane. In hyperdivergent, the mean difference is highest between 20° and parallel to the occlusal plane. There is no statistically significant difference between difference in angles of insertion.

7 mm

**Figure 9:**
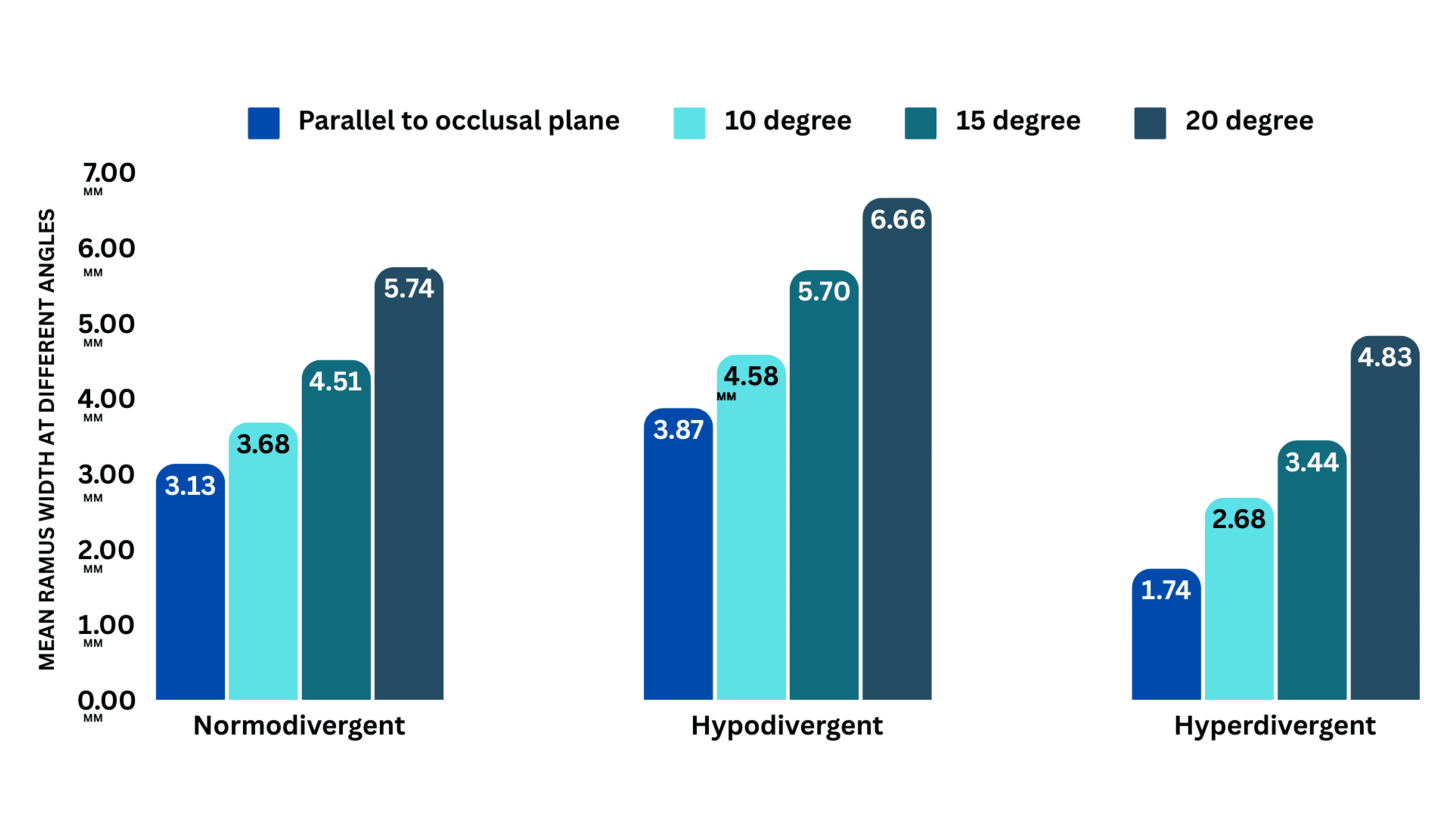
Intergroup comparison showing four angles of insertion in different growth patterns at 7 mm

There is a statistically significant difference (p < 0.05) in angles across growth patterns. The highest clearance at a 20° angle is normodivergent (5.74 ± 1.98), hypodivergent (6.66 ± 2.76), and hyperdivergent (4.83 ± 2.42) (Figure 9, Tables 7-8).

**Table 7:** Intergroup comparison of angle of insertion with different growth patterns for 7 mm

Growth pattern		Angle of insertion	N	Mean	Standard deviation	One way ANOVA	
						F	p-value
Normodivergent	7 mm	Parallel to the occlusal plane	10	3.13	1.99	2.67	0.067
		10 degree	10	3.68	2.329		
		15 degree	10	4.51	2.447		
		20 degree	10	5.74	1.988		
	Hypodivergent	7 mm	Parallel to the occlusal plane	10	3.87	2.299	2.5	0.075
		10 degree	10	4.58	2.119			
		15 degree	10	5.7	2.595			
		20 degree	10	6.66	2.762			
Hyperdivergent	7 mm	Parallel to the occlusal plane	10	1.74	1.779	3.56	0.024
		10 degree	10	2.68	2.092		
		15 degree	10	3.44	2.391		
		20 degree	10	4.83	2.428		

**Table 8:** Tukey’s post-hoc test for the intergroup comparison

	Growth pattern (I)	Growth pattern (J)	Mean difference (I-J)	p-value
Hyperdivergent	10 degree	15 degree	-0.76	0.864
	10 degree	20 degree	-2.15	0.143
	10 degree	Parallel to the occlusal plane	0.94	0.772
	15 degree	20 degree	-1.39	0.495
	15 degree	Parallel to the occlusal plane	1.7	0.32
	20 degree	Parallel to the occlusal plane	3.09	0.016
Hypodivergent	10 degree	15 degree	-1.12	0.739
	10 degree	20 degree	-2.08	0.249
	10 degree	Parallel to the occlusal plane	0.71	0.916
	15 degree	20 degree	-0.96	0.818
	15 degree	Parallel to the occlusal plane	1.83	0.356
	20 degree	Parallel to the occlusal plane	2.79	0.071
Normodivergent	10 degree	15 degree	-0.83	0.833
	10 degree	20 degree	-0.26	0.174
	10 degree	Parallel to the occlusal plane	0.55	0.943
	15 degree	20 degree	-1.23	0.599
	15 degree	Parallel to the occlusal plane	1.38	0.505
	20 degree	Parallel to the occlusal plane	2.61	0.055

Tukey’s post-hoc analysis shows the pairwise comparison of the angle of insertion with the other for 7 mm in different growth patterns. The groups are placed in two columns group (I) and group (J). The mean difference is seen in column (I-J). A negative value of mean difference indicates that the value in group (I) is less than that seen in group (J), and a positive value in the mean difference column suggests that the value in group (I) is more than that seen in group (J). In hypodivergent, the mean difference is highest between 20° and parallel to the occlusal plane. There is no statistically significant difference between difference in angles of insertion. In normodivergent, the mean difference is the highest between 20° and parallel to the occlusal plane. There is a statistically significant difference only between 20° and parallel to the occlusal plane. In hyperdivergent, the mean difference is highest between 20° and parallel to the occlusal plane. There is a statistically significant difference between the difference in 10° v/s 20° and 20° and parallel to the occlusal plane.

Clearance from the inferior alveolar canal decreases progressively from hypodivergent to normodivergent to hyperdivergent growth patterns at all levels.

## Discussion

The mandibular second molars have a relatively low incidence rate of impaction, and their management is considerably more challenging than that of other frequently impacted teeth.

Multiple methods exist for retrieving deeply impacted mandibular molars, employing both conventional and skeletal anchorage techniques, but the success rate of these methods varies [9]. Lin et al. reviewed six different methods for recovering deeply impacted molars and concluded that the most reliable and effective method involved surgically exposing the deeply impacted molars and uprighting them using a ramal bone screw [4]. The optimal force direction, i.e. occlusal and distal direction of force, is achieved when employing Ramal implants for the uprighting of mandibular second molars, facilitating early alignment of molars [25].

Anatomically, Ramal implants are placed into the anterior part of the ramus of the mandible, i.e., retromolar fossa, intraorally, situated medial to the external oblique ridge of the ramus intraorally, which can be identified by palpation. Before engaging the dense cortical bone, a Ramal bone screw needs to penetrate soft tissue that is thicker than that of a buccal shelf region, necessitating a long length of the transgingival collar in the ramal bone screw.

The intricate structure of the Ramal region leads to difficulty in the judgment of the location of the IAC as the Ramal area is narrow and the location of the inferior alveolar nerve is precise, which can lead to injury of IAN. This can be avoided by changing the angulations of screw placement; therefore, in this research investigation, two significant anatomical considerations essential for the successful placement of implants in the ramal region were analyzed, the maximal width at the transverse section of the ramus anteromedial border at three different heights from the occlusal plane (3, 5, and 7 mm above the central groove of the permanent mandibular first molar) in different growth patterns, and the proximity to the IAC when the ramal implant will be placed at three different heights and four different insertion angles (0°, 10°, 15°, and 20°), measured from a reference line that is parallel from the occlusal plane.

The findings of this study revealed that in individuals with a hypodivergent growth pattern, the highest average transverse width of the ramus was measured at 15.69 ± 0.952 mm, specifically at a height of 3 mm upward from the first molar of the mandibular arch.

The mean at 3 mm of all growth patterns is maximum, suggesting that as we move superiorly, the ramus width decreases. In addition, the ramus width decreases as the mandibular plane increases, signifying that the maximum width is seen in the hypodivergent growth pattern followed by normodivergent and hyperdivergent, signifying the difference in the anatomy of the mandible and morphology of the growth pattern.

The results suggest that a 2 mm diameter Ramal implant would possess adequate bone width for anchorage at all three positions assessed above the central groove of the permanent mandibular first molar (3, 5, and 7 mm). Clinical observations have demonstrated that positioning the implant at a higher level relative to the mandibular first molar provides the clinician with increased clearance to apply traction for disimpacting the impacted mandibular second molar [26].

Another factor we took into account was calculating the distance between the implant and the IAC while considering that the Ramal bone can only incorporate 5 mm of the implant [3]. Our study findings indicate that positioning Ramal implants maintains a safe distance from the IAC. This was observed through measurements taken at 3, 5, and 7 mm above the permanent mandibular first molar's central groove.

Among all three growth patterns, the highest clearance from IAC was observed at 20°.

It was observed from the result that the Ramal implant can be placed at 10° or 15° in a hypo-divergent growth pattern and at 15° in a normodivergent growth pattern. In a hyperdivergent growth pattern, a Ramal implant cannot be placed at an angle less than 20° due to its proximity to the IAC, which could potentially damage the neurovascular bundle. Thus, it is recommended to place the implant more laterally in case of a hyperdivergent growth pattern. In hypodivergent and normodivergent growth patterns, the Ramal implant can be positioned at 5 mm to 7 mm height from the mandibular first molar at a 15° or 20° angle. However, in hyperdivergent growth patterns, it is suggested to position it at an angle greater than 20° at 5 or 7 mm. The findings of this study were statistically significant.

The research done by Gaffuri et al. [36] revealed a statistically significant correlation between facial growth patterns and the height and thickness of alveolar bone. Specifically, the anterior portion of the maxilla and whole mandible exhibited thinner cortical bone in individuals with a hyperdivergent growth pattern. According to Ozdemir et al. [37], the hyperdivergent facial pattern group presented slightly narrower cortical bone than the hypodivergent one, suggesting that subjects with this facial type tend to have less dense buccal cortical bone in the maxillary and mandibular alveolar processes. Hence, limiting anteroposterior movements in hyperdivergent patients is advisable to minimize the risk of fenestration and dehiscence. Our study aligns with the above research, indicating that the transverse Ramal width is greatest in the hypodivergent growth pattern and smallest in the hyperdivergent growth pattern.

Our research results align with the findings of Maliael et al., who similarly determined that the transverse width of the ramus was greatest at 12.48 ± 1.76 mm, observed at a height of 3 mm above the mandibular first molar [26].

The research conducted by Patni J indicated that the average angle between the optimal site for Ramal implant placement and the occlusal line passing through the permanent mandibular first and second molars was 19.04° (SD ± 6.89). In addition, the proximity of the neurovascular bundle from the tip of the screw was measured at 7.1773 mm (SD ± 1.73988). This corroborates the outcomes of our research that the clearance from the IAC decreases progressively from hypodivergent to normodivergent to hyperdivergent growth patterns across all levels. They recommended lateral angulation of 13°-25° of the implant from the occlusal line to acquire a considerable amount of clearance of up to 7 mm from the IAC, which reduces the probability of its injury [27].

Our study's results parallel the observation of Mehta S et al. [38] stated that the optimal insertion site for mini-screws, determined based on sufficient ramus depth and thickness, was identified as 5 mm above the occlusal plane. Individuals with a hyperdivergent facial type exhibited significantly diminished ramus depth compared to those with hypodivergent and normodivergent facial types.

Taking into consideration the disparity in the IAC position in different individuals, the operators should employ a CBCT scan to plan the pathway of ramal implant insertion that averts any contact with the IAC.

This study is limited in assessing soft tissue thickness as it is different for every patient. It is dependent on the body type of the patient or any habit. Moreover, the effect of the patient's growth status and gender disparity needs to be studied.

This is pioneering research and the further scope of the study is that accurate implant placement can be done by stereolithography technique, guided implant placement, and virtual placement of an implant in CBCT. Tailoring implant angulations based on the patient's specific facial pattern ensures optimal biomechanical performance and reduces the risk of complications. Computer-aided design/computer-aided manufacturing (CAD/CAM) technology allows for the fabrication of patient-specific implants, enhancing adaptability to diverse facial patterns. Customized implants can accommodate variations in bone morphology and soft tissue thickness, promoting better aesthetic outcomes and functional stability. Clinical studies should also be carried out to assess the advantages and efficiency of the placement of ramal implants.

## Conclusions

Ramal implants can be securely placed 3-7 mm above the central groove of the permanent mandibular first molar across various growth patterns, with the optimal angulation being 15°-20° for hypodivergent and normodivergent patterns and 20° or more for hyperdivergent patterns to maintain adequate clearance from the inferior alveolar canal. The findings highlight the importance of evaluating skeletal parameters to ensure implant stability and minimize complications. These insights provide a practical framework for orthodontists, enhancing treatment precision and safety. Future research should validate these findings through advanced imaging and diverse population studies.
